# Supplementary figures and images for: Efficacy of Artificial Intelligence-Assisted Discrimination of Oral Cancerous Lesions from Normal Mucosa Based on the Oral Mucosal Image: A Systematic Review and Meta-Analysis
Source: Cancers (Basel). 2022 Jul 19;14(14):3499. doi: 10.3390/cancers14143499 (PMC9320189; doi:10.3390/cancers14143499)

## Sensitivity

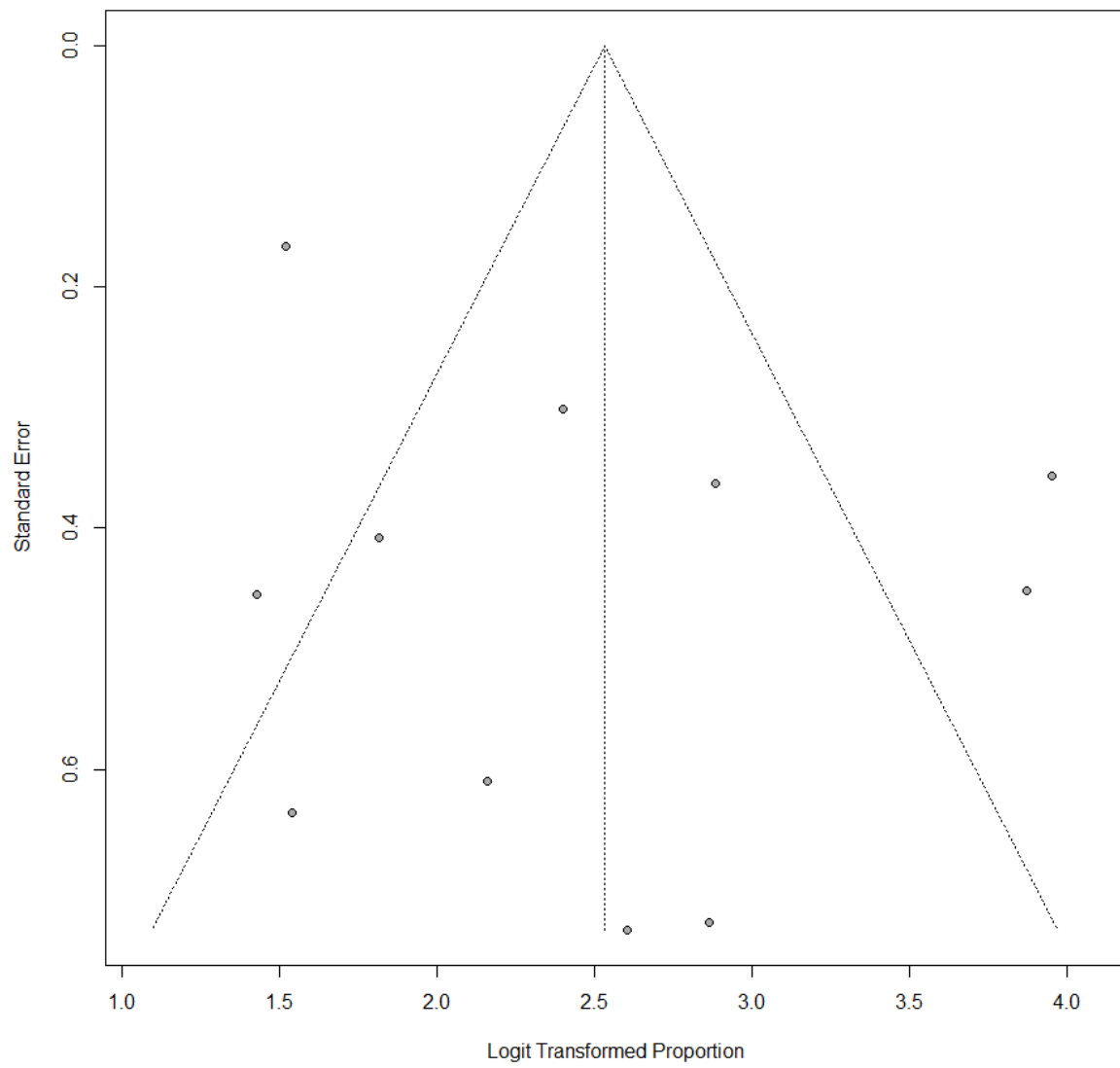

## Specificity

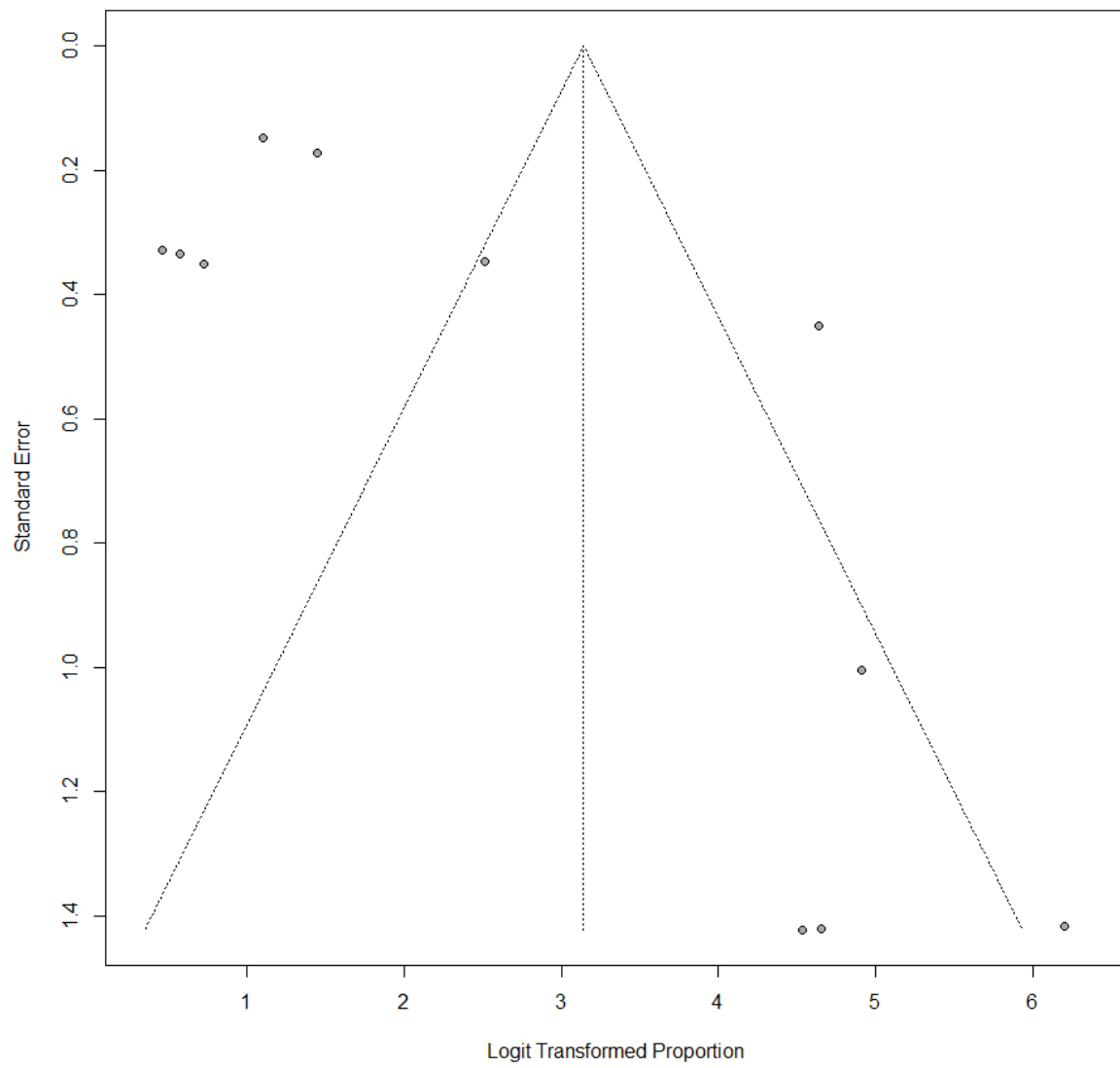

NPV

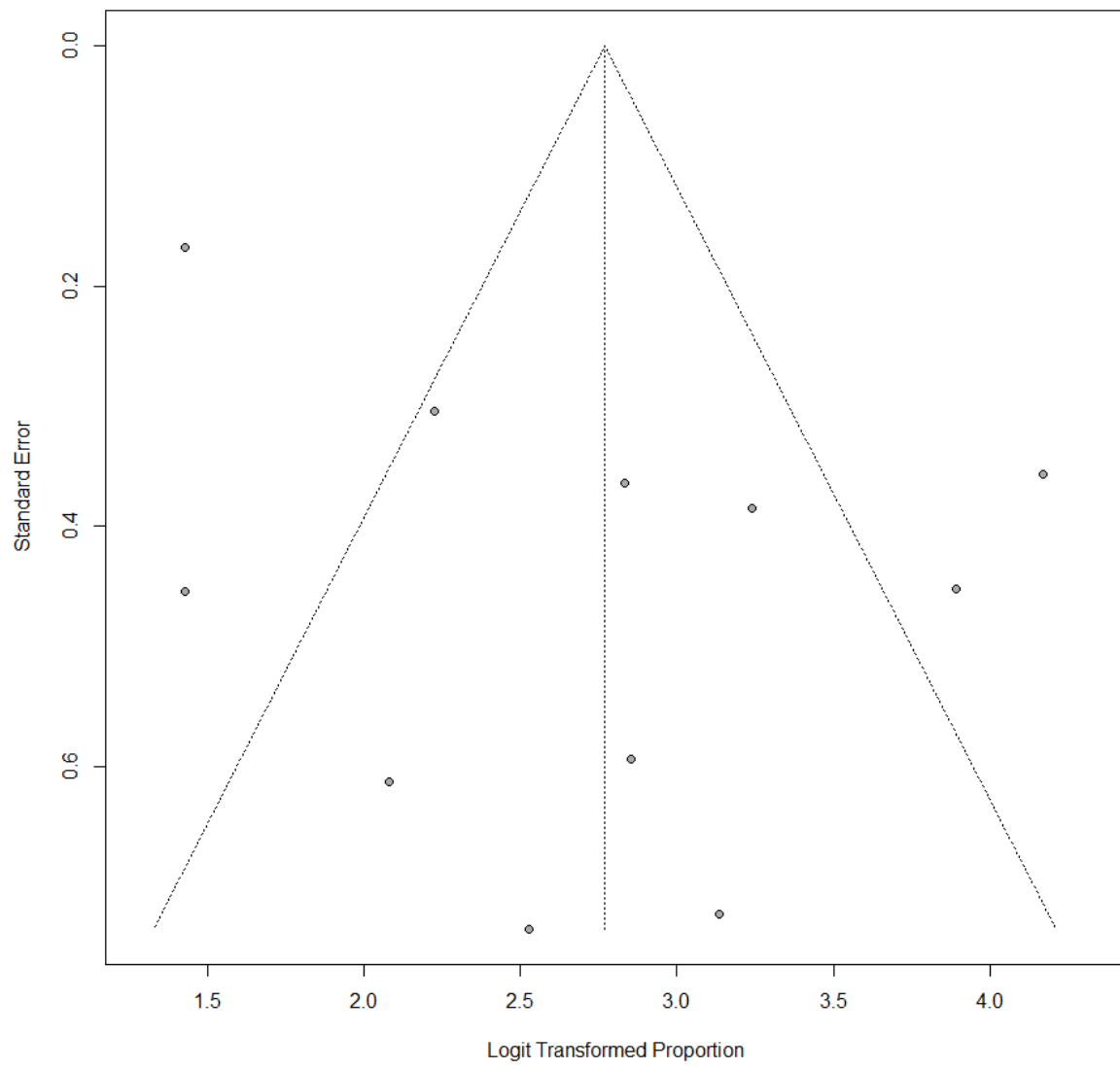

DOR

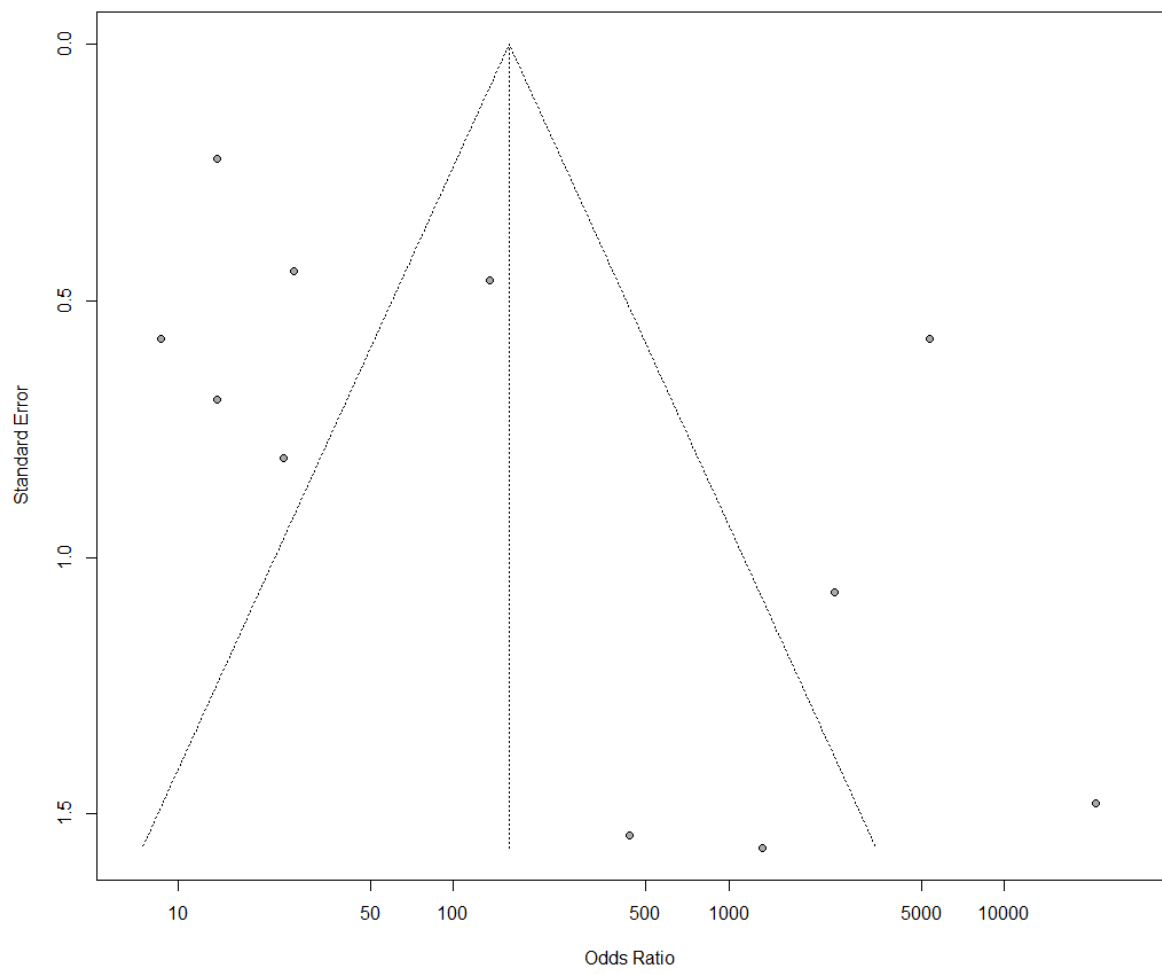

**Figure S1.** Begg's funnel plot.

Supplement: Supplementary file 1 [file cancers-14-03499-s001.zip › cancers-1816023-Supplementary figure.pdf]
